# Preference for On-Demand Fexuprazan Therapy in Gastroesophageal Reflux Disease: A Prospective Cohort Study

**DOI:** 10.3390/jpm15010019

**Published:** 2025-01-06

**Authors:** Byung Wook Jung, Chan Hyuk Park, Chang Soo Eun

**Affiliations:** 1 Department of Internal Medicine, Hanyang University Guri Hospital, Hanyang University College of Medicine, Guri 11923, Republic of Korea; 2 Department of Medicine, Graduate School, Yonsei University College of Medicine, Seoul 03722, Republic of Korea; 3 Department of Internal Medicine, Chung-Ang University H.C.S. Hyundae Hospital, Namyangju 12013, Republic of Korea

**Keywords:** fexuprazan, maintenance therapy, gastroesophageal reflux disease

## Abstract

**Introduction:** Maintenance therapy is crucial in managing and preventing symptom relapse in gastroesophageal reflux disease (GERD), with continuous and on-demand therapy being the common approaches. However, maintenance therapy using potassium-competitive acid blockers (P-CABs), such as fexuprazan, remains incompletely evaluated. **Methods:** This single-center, single-arm, prospective cohort study enrolled individuals with weekly heartburn or acid regurgitation and confirmed erosive esophagitis. The participants received 40 mg fexuprazan daily for 4 weeks as initial therapy, followed by 4 weeks of maintenance therapy. Patients chose either continuous or on-demand therapy for maintenance, according to their preference. The primary endpoint was the proportion of patients selecting on-demand therapy. The symptom scores were assessed using the GERD questionnaire (GERD-Q) and patient assessment of upper-gastrointestinal-disorders symptoms questionnaire (PAGI-SYM). **Results:** The 31 included participants showed a significant reduction in symptom scores after initial treatment (baseline vs. 4-week: GERD-Q, 9.0 vs. 6.5, *p* < 0.001; PAGI-SYM, 29.0 vs. 10.8, *p* < 0.001). Twenty-one (67.7%) patients chose on-demand therapy after initial treatment. The symptom scores did not differ significantly before and after maintenance therapy (4-week vs. 8-week: GERD-Q, 6.5 vs. 6.0, *p* = 0.225; PAGI-SYM, 10.8 vs. 9.0, *p* = 0.354). Although this relation was not significant, patients experiencing larger decreases in symptom scores tended to prefer on-demand therapy. After maintenance therapy, the symptom scores did not differ between continuous and on-demand therapy (GERD-Q, 5.3 vs. 6.3, *p* = 0.342; PAGI-SYM, 9.4 vs. 8.8, *p* = 0.611). **Conclusions:** Fexuprazan was effective as an initial and maintenance therapy in patients with GERD who showed typical symptoms. Approximately 68% of the patients preferred on-demand therapy as a maintenance treatment. Based on the patient’s preference for maintenance therapy, symptom control did not differ between continuous and on-demand therapy.

## 1. Introduction

Gastroesophageal reflux disease (GERD) is a chronic condition characterized by relapse in approximately 50–80% of patients despite achieving symptom control and mucosal healing using acid-suppressants, such as proton pump inhibitors (PPIs) [1]. Typically, long-term maintenance therapy is required to minimize GERD symptom relapse, even after 4 or 8 weeks of initial treatment [2]; various approaches have been explored, including continuous, intermittent, and on-demand therapy. Continuous therapy involves the daily intake of PPIs, whereas on-demand therapy entails using PPIs solely when symptoms arise [1].

Continuous therapy may be the most effective form of maintenance treatment for preventing symptom recurrence, as it involves taking medication regularly. However, some patients have challenges with continuous therapy due to the inconvenience of daily medication intake. An internet survey reported that 57.6% of patients with GERD expressed a desire for maintenance treatment, with 76.1% preferring on-demand therapy [1]. Medication adherence declines abruptly during the first 6 months of PPI therapy among long-term users [3]. According to a meta-analysis conducted for the 2020 Seoul Consensus Guidelines, symptom control did not differ significantly between continuous and on-demand therapy among patients with mild erosive esophagitis or non-erosive reflux disease [1]. Therefore, on-demand therapy may be a sufficient option for patients with non-severe GERD.

However, most of the previous research focused on PPI-based initial and maintenance therapies [4,5,6,7,8,9]. Whether these findings can be extrapolated to the use of a novel class of acid-suppressing agents, called potassium-competitive acid blockers (P-CABs), in the maintenance therapy of GERD remains unclear. P-CABs offer a more potent and long-lasting effect than PPIs, making them promising options for the initial and maintenance treatment of GERD [1,10]. Fexuprazan, in particular, is a new P-CAB characterized by a rapid onset of action and mean elimination half-life of up to 9 h, highlighting its prolonged duration of action. In the present study, we aimed to investigate the preferences for maintenance therapy among patients with GERD who underwent initial treatment with fexuprazan, and assess the effectiveness of such maintenance therapy. If the majority of GERD patients who received initial treatment with fexuprazan prefer on-demand therapy for maintenance and find it effective, we may actively consider prescribing fexuprazan as an initial treatment and subsequently recommending on-demand therapy for GERD patients in the future.

## 2. Methods

### 2.1. Study Design

This single-center, single-arm, prospective cohort study was conducted at Hanyang University Guri Hospital between August 2022 and June 2024. The inclusion criteria were individuals aged ≥19 years, experiencing heartburn or acid regurgitation at least once a week, and having a confirmed diagnosis of erosive esophagitis. Patients who had undergone surgical procedures on the esophagus, stomach, or duodenum were excluded. This study was approved by the Institutional Review Board on Human Subjects Research and Ethics Committee of Hanyang University Guri Hospital (IRB no: GURI 2022-03-025; date of approval 23 May 2022) and was registered at the Clinical Research Information Service (CRIS registration number: KCT0008526).

### 2.2. Initial Treatment and Maintenance Therapy

Demographic information such as age and gender, along with height, weight, smoking history, and alcohol consumption were collected for participants who agreed to enroll in the study. Additionally, two symptom evaluation questionnaires (GERD questionnaire [GERD-Q] and patient assessment of upper-gastrointestinal-disorders symptoms questionnaire [PAGI-SYM]) were completed through structured interviews [11,12].

Participants received 40 mg fexuprazan (Fexuclue^®^, Daewoong Pharm. Co., Ltd., Seoul, Republic of Korea) once daily for 4 weeks as initial treatment, taken after breakfast. Subsequently, symptom evaluation questionnaires were administered to assess the effectiveness of the initial treatment. Then, maintenance therapy was administered for 4 weeks, during which the patients were allowed to continue taking 40 mg fexuprazan once daily or only when needed, according to their preference. After this 4-week maintenance period, the number of remaining medications was checked to verify the actual intake, and symptom evaluation questionnaires were administered again.

The patients who consumed ≥80% of the medication during the maintenance therapy period were considered to have chosen continuous therapy, whereas those who consumed <80% were considered to have chosen on-demand therapy.

### 2.3. Study Endpoint and Measurements

The primary endpoint of this study was the proportion of patients selecting on-demand therapy. The secondary endpoint included the efficacy of initial and maintenance therapy, and the comparative efficacy between patients selecting continuous therapy and those selecting on-demand therapy.

The results of the GERD-Q were analyzed together with the total and frequency scores of typical symptoms (heartburn or acid regurgitation) [11]. For the PAGI-SYM, along with the total scores, the following six subscales were analyzed: nausea/vomiting, fullness/early satiety, bloating, upper abdominal pain, lower abdominal pain, and heartburn/regurgitation [12].

### 2.4. Sample Size Calculation

The sample size was calculated considering that approximately 75% of patients preferred on-demand therapy during maintenance therapy with PPIs [1]. To estimate the proportion with a confidence interval width of <0.3 at a 95% confidence level, 30 participants were needed. Allowing for a 5% dropout rate, a sample size of 32 participants was determined for recruitment.

### 2.5. Statistical Analysis

Continuous variables are presented as means with the associated standard deviation (SD) and were compared using paired *t*-tests. The Mann–Whitney U test was performed to compare independent groups where non-parametric testing was required. Categorical variables are presented as numbers with proportions. The reported *p*-values were assessed using a two-sided test, and statistical significance was set at *p* < 0.05. All statistical analyses were performed using R statistical software (version 4.3.0; R Foundation for Statistical Computing, Vienna, Austria).

## 3. Results

### 3.1. Study Participants and Baseline Characteristics

**Figure 1:**
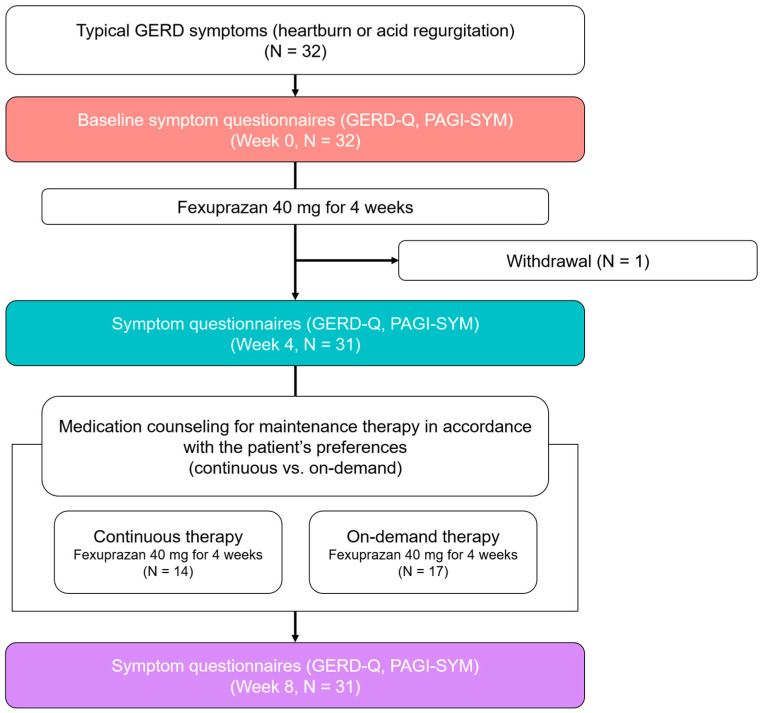
Study flow diagram. On-demand therapy was defined as consuming <80% of prescribed medication. GERD-Q, gastroesophageal reflux disease questionnaire; PAGI-SYM, patient assessment of upper-gastrointestinal-disorders symptoms questionnaire.

The study flow diagram is shown in Figure 1. This study included 32 patients. No patient was excluded based on the exclusion criteria. All the patients completed the GERD-Q and PAGI-SYM and were prescribed 40 mg fexuprazan for 4 weeks as initial treatment. Subsequently, one patient withdrew from the study, leaving 31 patients who completed 4 weeks of initial treatment for GERD. Following initial treatment, symptom evaluation was performed using the GERD-Q and PAGI-SYM. Thereafter, the patients were counseled to continue taking the medication regularly or take it only when needed according to their preference for the next 4 weeks. Consequently, 10 (32.3%) and 21 (67.7%) patients chose continuous and on-demand therapy, respectively. The actual medication adherence rate was 88.9% (SD 6.8%) in the continuous therapy group and 48.8% (SD 21.5%) in the on-demand therapy group. Symptom evaluation was conducted again after maintenance therapy using the GERD-Q and PAGI-SYM.

Table 1 presents the baseline characteristics of the study participants. The mean age of participants was 54 years, with 51.6% being male. The mean body mass index was 25.3 ± 4.4 kg/m^2^. Table 2 shows the baseline symptom scores. The baseline GERD-Q and PAGI-SYM scores were 9.0 ± 2.3 and 29.0 ± 18.7, respectively.

### 3.2. Treatment Response

Table 2 describes the treatment response after initial and maintenance therapy. Following initial therapy, the GERD-Q total score decreased significantly from 9.0 ± 2.3 to 6.5 ± 1.9, and the heartburn and regurgitation scores also decreased significantly. Similarly, the PAGI-SYM total score decreased from 29.0 ± 18.7 to 10.8 ± 12.2 points after initial therapy, with significant score reductions observed in the nausea/vomiting, fullness/early satiety, bloating, upper abdominal pain, and heartburn/regurgitation subscales. Although the lower abdominal pain subscale decreased from 1.0 to 0.4 points, the difference was not statistically significant.

After maintenance therapy, significant reductions in the symptom scores were observed in all items compared with baseline. However, after maintenance therapy, the GERD-Q and PAGI-SYM total scores were 6.0 ± 2.1 and 9.0 ± 9.3 points, respectively, with no significant difference compared to before maintenance therapy.

**Figure 2:**
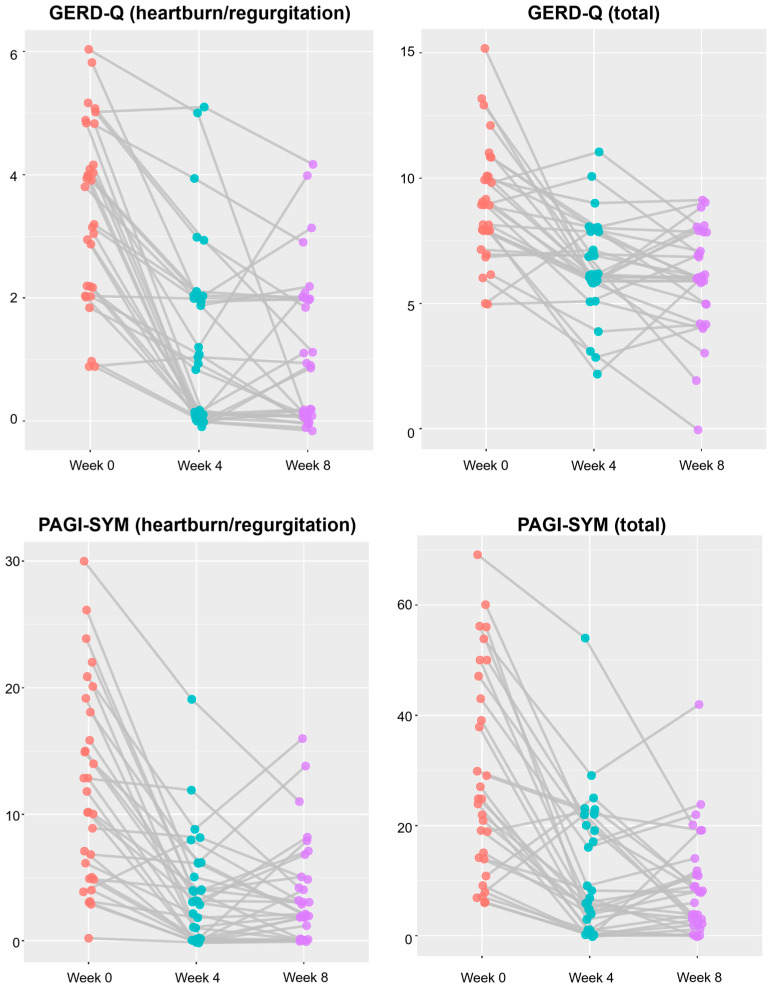
Changes in GERD-Q and PAGI-SYM scores. Each dot represents a study participant, with orange indicating the baseline, cyan representing the 4-week point, and purple denoting the 8-week point. GERD-Q, gastroesophageal reflux disease questionnaire; PAGI-SYM, patient assessment of upper-gastrointestinal-disorders symptoms questionnaire.

Figure 2 shows the further changes in the symptom scores. In most patients, the symptom scores decreased after initial therapy, whereas they were relatively well-maintained during maintenance therapy.

No adverse events were observed throughout the entire duration of initial and maintenance therapy.

### 3.3. Preference for Maintenance Therapy

**Figure 3:**
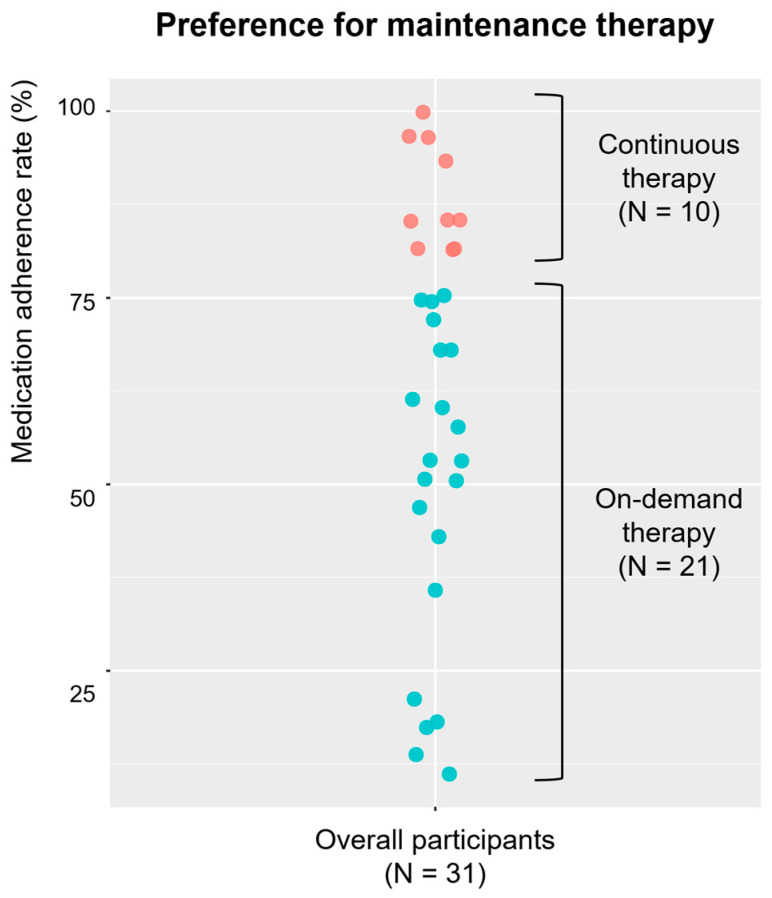
Medication adherence rate during maintenance therapy. Continuous and on-demand therapies were allowed during maintenance therapy. On-demand therapy was defined as consuming <80% of prescribed medication. Each dot represents a study participant, with orange indicating those who received continuous therapy and cyan representing those who received on-demand therapy.

Figure 3 depicts the medication adherence of patients during the maintenance therapy. Of the 31 patients, 10 were classified into the continuous therapy group, whereas the remaining 21 patients were in the on-demand therapy group. Table 3 demonstrates whether the symptom scores were different between the continuous and on-demand therapy groups at baseline, after initial therapy, and after maintenance therapy. The symptom scores were not significantly different between the continuous and on-demand therapy groups at baseline and after initial therapy, suggesting that the symptom scores did not strongly influence the choice of maintenance therapy. Furthermore, the symptom scores were not significantly different between the continuous and on-demand therapy groups after maintenance therapy. This indicates that both continuous and on-demand therapy proved effective as maintenance therapies.

**Figure 4:**
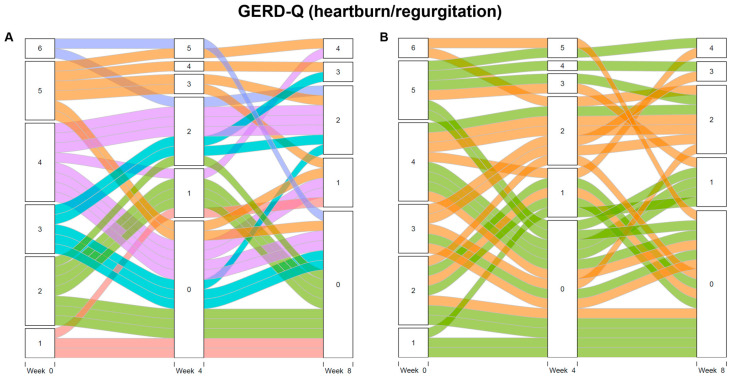
Changes in heartburn and regurgitation frequency scores of GERD-Q. Left panel (**A**) is color-coded based on baseline scores (range: 0–6); right panel (**B**) is color-coded based on preference for maintenance therapy. In the left panel, the different colors represent the GERD-Q scores at the baseline. In the right panel, orange represents continuous therapy and green represents on-demand therapy. Y-axis indicates heartburn and regurgitation frequency scores. GERD-Q, gastroesophageal reflux disease questionnaire.

To further explore the impact of symptom changes on the choice of maintenance therapy, an alluvial diagram is presented in Figure 4. Although no statistically significant differences were observed between the continuous and on-demand therapy groups in the previous analysis, the alluvial diagram reveals a tendency for patients who experienced relatively large decreases in symptom scores from baseline after initial therapy to choose on-demand therapy.

## 4. Discussion

This study confirmed the effectiveness of fexuprazan as an initial and maintenance treatment for patients with GERD. Although this study was planned as a single-arm study, and thus cannot be compared with other studies exploring different medications, previous randomized controlled trials (RCTs) have shown that fexuprazan is as effective as PPIs in treating erosive esophagitis [13,14]. However, unlike previous RCTs, this study focused on assessing the improvements in symptoms. Significant improvements were observed in typical symptoms, such as heartburn and regurgitation (measured using the GERD-Q) and the heartburn/regurgitation subscale of PAGI-SYM. Interestingly, other than the typical symptoms, such as heartburn and regurgitation, improvements were also observed in other subscales associated with atypical or dyspepsia-related symptoms, such as nausea/vomiting, fullness/early satiety, bloating, and upper abdominal pain, suggesting that fexuprazan may also improve the symptoms of patients with overlapping functional dyspepsia and GERD.

Approximately 68% of the patients who received initial therapy with fexuprazan for 4 weeks preferred on-demand therapy, indicating that many patients saw no need for continuous medication intake due to excellent symptom improvement. Furthermore, continuous and on-demand therapy after the initial therapy proved effective in maintaining symptom control without worsening symptoms. This was observed in the heartburn/regurgitation scales of the GERD-Q and PAGI-SYM and in several dyspepsia-related subscales.

The baseline symptom scores or symptom scores after initial therapy were not significantly different between patients choosing continuous therapy and those choosing on-demand therapy, making it difficult to clearly identify which patients preferred on-demand therapy. However, the alluvial diagram (Figure 4) suggests a tendency for patients who responded better to initial therapy to choose on-demand therapy. Therefore, we speculated that controlling symptoms well initially helps minimize medication use while maintaining patient satisfaction.

This study agrees with previous guidelines suggesting that on-demand therapy may be a good option for patients with non-severe GERD [1]. Although recommendations were previously based on studies involving PPIs, on-demand therapy may also be a feasible and practical option in treatments with P-CABs. Notably, many studies have raised concerns regarding the association between long-term PPI use and adverse events, including bone fracture, micronutrient deficiency, and *Clostridioides difficile*-associated colitis [15,16,17,18]. Even if the causal relationship has not been established, several patients are concerned about long-term adverse events resulting from acid-suppressive agents. The best clinical practice is prescribing the minimal necessary medication. Therefore, the use of on-demand therapy in the maintenance treatment of GERD may be emphasized.

Although our study, to the best of our knowledge, is the first to investigate preferences for maintenance therapy with fexuprazan in patients with GERD, some limitations must be considered. First, as a single-arm study, it was challenging to fully compare the effectiveness of fexuprazan as initial and maintenance therapy with that of PPIs. However, our study primarily aimed to assess patient preferences during maintenance therapy, and the efficacy of fexuprazan compared with that of PPIs has already been established in other studies [13,14]. Second, the comparison of symptom scores between the group choosing continuous therapy and the one choosing on-demand therapy may have lacked sufficient statistical power due to the small number of patients in each group. Although several patients opted for on-demand therapy in our study, further research with a larger sample size is needed to compare symptom scores between on-demand and continuous therapy more definitively. Finally, the duration of maintenance therapy was relatively short. Future studies should investigate whether a preference for on-demand therapy persists when maintenance therapy is extended beyond 6 months.

## 5. Conclusions

Despite these limitations, our study provides a better understanding of patient preferences and the effectiveness of on-demand therapy with fexuprazan. In patients with GERD who showed typical symptoms, 4 weeks of fexuprazan therapy demonstrated reduced GERD and dyspepsia-related symptoms. Moreover, more than half of the patients opted for on-demand therapy as a maintenance treatment, and the symptom scores between patients choosing on-demand therapy and those choosing continuous therapy were not significantly different.

## Figures and Tables

**Table 1:** Baseline characteristics of included patients.

Variable	Value
Number of patients, n	31
Age, mean ± SD	54.4 ± 15.0
Sex, n (%)	
Male	16 (51.6)
Female	15 (48.4)
BMI, mean ± SD	25.3 ± 4.4
Smoking habit, n (%)	
Non-smoker	15 (48.4)
Former smoker	6 (19.4)
Current smoker	10 (32.3)
Alcohol consumption, n (%)	
Non-drinker	14 (45.2)
Former drinker	2 (6.5)
Current drinker	15 (48.4)

SD, standard deviation.

**Table 2:** Baseline symptoms and treatment response.

Variable		Baseline (Week 0)	Initial Therapy (Week 4)		Maintenance Therapy (Week 8)		
		Score	Score	*p*-Value (vs. Baseline)	Score	*p*-Value (vs. Baseline)	*p*-Value (vs. Week 4)
GERD-Q, mean ± SD							
	Heartburn frequency score (0–3)	1.9 ± 1.0	0.6 ± 0.8	<0.001	0.5 ± 0.8	<0.001	0.768
	Regurgitation frequency score (0–3)	1.5 ± 1.0	0.7 ± 0.9	<0.001	0.5 ± 0.8	<0.001	0.344
	Total score (0–18)	9.0 ± 2.3	6.5 ± 1.9	<0.001	6.0 ± 2.1	<0.001	0.225
PAGI-SYM, mean ± SD							
	Nausea/vomiting (0–15)	3.2 ± 3.1	1.0 ± 1.8	<0.001	1.3 ± 3.5	0.010	0.740
	Fullness/early satiety (0–20)	6.6 ± 4.6	3.5 ± 3.9	<0.001	2.4 ± 2.5	<0.001	0.091
	Bloating (0–10)	2.7 ± 2.9	0.8 ± 1.5	0.001	0.9 ± 1.6	0.001	0.837
	Upper abdominal pain (0–10)	3.8 ± 2.9	1.4 ± 2.2	<0.001	0.9 ± 1.2	<0.001	0.194
	Lower abdominal pain (0–10)	1.0 ± 1.7	0.4 ± 1.0	0.089	0.3 ± 0.7	0.027	0.502
	Heartburn/regurgitation (0–35)	11.9 ± 7.8	3.6 ± 4.3	<0.001	3.7 ± 4.1	<0.001	0.893
	Total score (0–100)	29.0 ± 18.7	10.8 ± 12.2	<0.001	9.0 ± 9.3	<0.001	0.354

**Table 3:** Association between treatment response and preference for maintenance therapy.

Variable	Continuous Therapy (N = 10)	On-Demand Therapy (N = 21)	*p*-Value
**At baseline (week 0)**			
GERD-Q, mean ± SD			
Heartburn frequency score (0–3)	1.7 ± 1.3	2.0 ± 0.9	0.757
Regurgitation frequency score (0–3)	1.9 ± 0.7	1.4 ± 1.0	0.177
Total score (0–18)	9.8 ± 2.8	8.7 ± 2.1	0.326
PAGI-SYM, mean ± SD			
Nausea/vomiting (0–15)	3.6 ± 3.9	3.0 ± 2.8	0.779
Fullness/early satiety (0–20)	7.8 ± 5.9	6.0 ± 4.0	0.552
Bloating (0–10)	2.6 ± 3.4	2.7 ± 2.8	0.727
Upper abdominal pain (0–10)	3.8 ± 3.3	3.9 ± 2.8	0.865
Lower abdominal pain (0–10)	1.4 ± 2.1	0.8 ± 1.5	0.429
Heartburn/regurgitation (0–35)	13.2 ± 7.5	11.3 ± 8.0	0.472
Total score (0–100)	32.4 ± 21.3	27.4 ± 17.6	0.627
**After initial therapy (week 4)**			
GERD-Q, mean ± SD			
Heartburn frequency score (0–3)	0.6 ± 0.8	0.6 ± 0.9	0.884
Regurgitation frequency score (0–3)	1.2 ± 1.1	0.4 ± 0.7	0.049
Total score (0–18)	6.3 ± 2.2	6.5 ± 1.9	0.615
PAGI-SYM, mean ± SD			
Nausea/vomiting (0–15)	1.4 ± 1.6	0.9 ± 1.9	0.088
Fullness/early satiety (0–20)	4.7 ± 2.9	3.0 ± 4.3	0.149
Bloating (0–10)	1.5 ± 2.1	0.5 ± 1.0	0.206
Upper abdominal pain (0–10)	2.1 ± 1.7	1.0 ± 2.3	0.034
Lower abdominal pain (0–10)	0.2 ± 0.6	0.5 ± 1.1	0.373
Heartburn/regurgitation (0–35)	6.0 ± 3.4	2.5 ± 4.2	0.004
Total score (0–100)	15.9 ± 7.9	8.4 ± 13.3	0.021
**After maintenance therapy (week 8)**			
GERD-Q, mean ± SD			
Heartburn frequency score (0–3)	0.4 ± 0.7	0.6 ± 0.8	0.466
Regurgitation frequency score (0–3)	0.6 ± 1.0	0.5 ± 0.7	0.883
Total score (0–18)	5.3 ± 2.7	6.3 ± 1.7	0.342
PAGI-SYM, mean ± SD			
Nausea/vomiting (0–15)	0.6 ± 1.1	1.6 ± 4.2	0.818
Fullness/early satiety (0–20)	2.7 ± 2.7	2.2 ± 2.4	0.681
Bloating (0–10)	0.7 ± 1.9	1.0 ± 1.5	0.191
Upper abdominal pain (0–10)	0.9 ± 1.1	1.0 ± 1.2	>0.999
Lower abdominal pain (0–10)	0.0 ± 0.0	0.4 ± 0.9	0.099
Heartburn/regurgitation (0–35)	4.5 ± 4.7	3.4 ± 3.9	0.379
Total score (0–100)	9.4 ± 8.2	8.8 ± 9.9	0.611

Continuous therapy was determined based on adherence to medication of 80% or higher. GERD-Q, gastroesophageal reflux disease questionnaire; PAGI-SYM, patient assessment of upper-gastrointestinal-disorders symptoms questionnaire; SD, standard deviation.

## Data Availability

All relevant data are available in the manuscript.
